# Supplementary material for: The Prognostic Value of Neutrophil-to-Lymphocyte Ratio in Patients with Metastatic Renal Cell Carcinoma
Source: Curr Oncol. 2023 Feb 17;30(2):2457–64. doi: 10.3390/curroncol30020187 (PMC9955537; doi:10.3390/curroncol30020187)
Supplement: Supplementary file 1 [file curroncol-30-00187-s001.zip › curroncol-2222850-supplementary.pdf]

		All<br>n(%)	Patient who<br>died	Not died	Log-Rank<br>P value	
		**median survival (IQR)**<br>**mean survival** ± SD (**range**)				
Gender	M	52(70.3)<br>8(5-14.50)<br>13.85±16.79<br>(1-84)	M 16(30.8)<br>4.50(3-8)<br>5.50±3.28<br>(1-13)	M 36(69.2)<br>11(6-23.75)<br>17.56±18.98<br>(3-84)	0.355	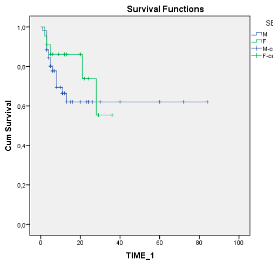
	F	22(29.7)<br>12.50(5-22.50)<br>14.95±10.06<br>(2-36)	F 5(29.7)<br>5.00(2.50-24.50)<br>11.80±11.90<br>(2-28)	F 17(77.3)<br>13(7.50-23)<br>15.88±9.66<br>(3-36)		
		0.774	0.304	0.734		
Location	S	34(45.9)<br>8(4-15.25)<br>13.91±17.99<br>(1-84)	S 11(32.4)<br>4(3-8)<br>4.91±3.47<br>(1-13)	S 23(67.6)<br>12(5-20)<br>18.22±20.50<br>(3-84)	0.352	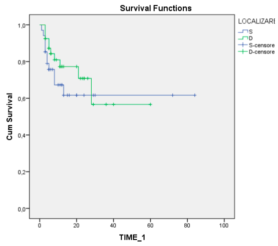
	D	40(54.1)<br>11(5.25-22.75)<br>14.40±12.24<br>(3-60)	D 10(25.0)<br>5.50(3-13.50)<br>9.30±8.55<br>3-28	D 30(75.0)<br>11.50(6-24)<br>16.10±12.92<br>(3-60)		
		0.891	0.157	0.648		
Fuhrman	2	2 35(47.3)<br>9(5-24)<br>14.34±13.87<br>(3-72)	2 7(20.0)<br>6(3-8)<br>8.71±8.75<br>(3-28)	2 28(80.0)<br>17.50(6-24)<br>15.75±14.66<br>(3-72)	0.085	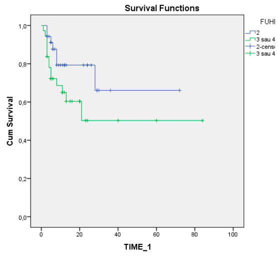
	3<br>sau<br>4	37(41.9)<br>11(4-18)<br>13.84±16.57<br>(1-84)	3 sau 4 14(37.8)<br>4(3-8.75)<br>6.14±5.47<br>(1-21)	3 sau 4 23(62.2)<br>13(6-20)<br>18.52±19.26<br>(3-84)		
		0.889	0.496	0.573		
Karnofsky	0	57(77.0)<br>12(6-21.50)<br>16.37±15.92<br>(3-84)	9(15.8)<br>8(4-12)<br>8.89±5.64<br>(3-21)	48(84.2)<br>12(6.25-23.75)<br>17.77±16.85<br>(3-84)	0.000	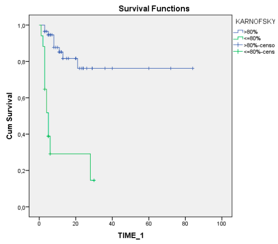
	1	17(23.0)<br>4(3-5.50)<br>6.82±8.46<br>(1-30)	12(70.6)<br>3.50(3-5)<br>5.58±7.19<br>(1-28)	5(29.4)<br>5(4-18)<br>9.80±11.34<br>(3-30)		
		0.021	0.269	0.308		

TTD	0	22(29.7)	2(9.1)	20(90.9)	0.009	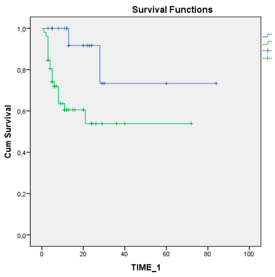
		13(7.25-25)	20.50(13-20.50)	12.50(5.75-23.75)		
		19.86±19.17	20.50±10.60	19.80±20.01		
		(3-84)	(13-28)	(3-84)		
	1	52(70.3)	19(36.5)	33(63.5)		
		7.50(4-14.50)	4(3-8)	11(6-22)		
		11.77±12.36	5.58±4.50	15.33±14.02		
		(1-72)	(1-21)	(3-72)		
		0.034	0.001	0.344		
Calcium	0	58(78.4)	15(25.9)	43(74.1)	0.595	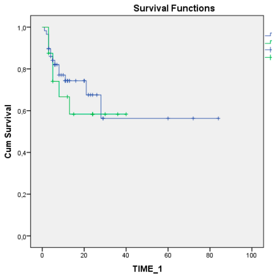
		9.50(5-17)	4.00(3-8)	11.00(6-20)		
		13.78±15.76	7.33±7.56	16.02±17.27		
		(1-84)	(1-28)	(3-84)		
	1	16(21.6)	6(37.5)	10(62.5)		
		12.50(5-24)	5.00(3-9.25)	24.00(10.20-31.50)		
		15.63±12.44	6.17±3.81	21.30±12.42		
		(3-40)	(3-13)	(3-40)		
		0.667	0.066	0.004		
HB	0	33(44.6)	7(21.2)	26(78.8)	0.002	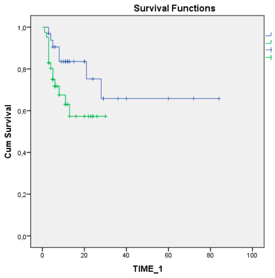
		12(8-26)	8(4-21)	12.50(10.75-29.00)		
		19.67±19.52	11.00±9.62	22.00±20.94		
		(3-84)	(3-28)	(3-84)		
	1	41(55.4)	14(34.1)	27(65.9)		
		6(4-13)	3.50(3.00-6.50)	8(5-22)		
		9.76±7.98	5.00±3.46	12.22±8.57		
		(1-30)	(1-13)	(3-30)		
		0.009	0.048	0.034		
LDH	0	62(83.8)	14(22.6)	48(77.4)	0.002	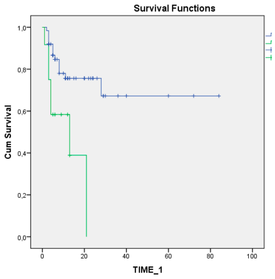
		11(5-22.25)	5(3-8)	12(6-24)		
		15.40±15.98	7.00±6.57	17.85±17.10		
		(2-84)	(2-28)	(3-84)		
	1	12(16.2)	7(58.3)	5(41.7)		
		5.50(3.25-12.75)	4(3-13)	9(5.50-12.50)		
		7.83±5.87	7.00±7.28	9.00±3.53		
		(1-21)	(1-21)	(5-13)		
		0.084	1.000	0.257		
Platelets	0	50(67.6)	12(24.0)	38(76.0)	0.075	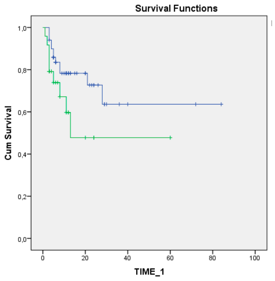
		11(5-22.25)	5(3.25-8)	12.50(6-24)		
		15.96±15.96	8.17±7.96	18.42±17.10		
		(3-84)	(3-28)	(3-84)		
	1	24(32.4)	9(37.5)	15(62.5)		
		6.50(3-12)	3(2.50-9.50)	8(4-20)		
		10.46±12.44	5.44±4.24	13.47±14.77		
		(1-60)	(1-13)	(3-60)		

		0.142	0.365	0.329		
neutrophil	0	62(83.8)	16(25.8)	46(74.2)	0.102	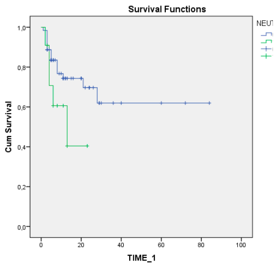
NA 1(1.4)		11(5-21.25)	5(3-8)	12(6-24)		
		15.23±16.06	7.38±7.29	17.96±17.38		
		(1-84)	(1-28)	(3-84)		
	1	11(14.9)	5(45.5)	6(54.5)		
		6(4-13)	4(3-9.50)	9.50(5.25-15.50)		
		8.45±6.19	5.80±4.27	10.67±7.01		
		(2-23)	(2-13)	(1-23)		
		0.174	0.655	0.318		
NLR	<3	45(61.6)	9(20.0)	36(80.0)	0.001	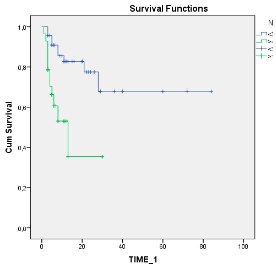
NA 1(1.4)		13(7-24)	8(4-16)	15.50(10.25-24)		
		18.87±17.26	10.22±8.67	21.03±18.26		
		(3-84)	(3-28)	(3-84)		
	>=3	28(38.4)	12(42.9)	16(57.1)		
		5(3-8)	3.50(3-5.75)	6(5-11)		
		6.71±5.68	4.58±3.23	8.31±6.64		
		(1-30)	(1-13)	(3-30)		
		0.000	0.058	0.001		
IMDC	fav.	5(6.8)	-	5(100)	0.000	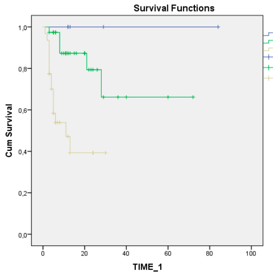
		13(12-56.50)	-	13(12-56.50)		
		30.00±31.04	-	30.00±31.04		
		(12-84)	-	(12-84)		
	inter	38(51.4)	6(15.8)	32(84.2)		
		11.50(7.50-22.25)	8(6.75-22.75)	12(6.75-22.75)		
		17.13±14.88	12.67±9.62	17.97±15.65		
		(3-72)	(3-28)	(3-72)		
	poor	31(41.9)	15(48.4)	16(51.6)		
		5(3-11)	4(3-5)	6.50(4.25-21.25)		
		8±7.56	4.73±3.24	11.06±9.16		
		(1-30)	(1-13)	(3-50)		
		0.002	0.009	0.068		
MSKCC	low	8(10.8)	-	8(100)	0.000	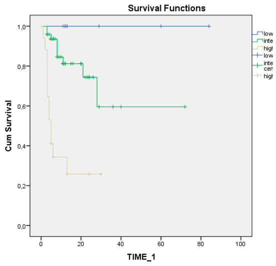
		13(12-52.25)	-	13(12-52.25)		
		29.25±27.77	-	29.25±27.77		
		(11-84)	-	(11-84)		
	inter	49(66.2)	9(18.4)	40(81.6)		
		11(5-20.50)	8(4-16)	11(5.25-21.50)		
		13.98±12.54	10.56±8.50	14.75±13.25		
		(3-72)	(3-28)	(3-72)		
	high	17(23.0)	12(70.6)	5(29.4)		
		5(3-9.50)	3.50(3-5)	13(5.50-27)		
		7.65±8.06	4.33±3.05	15.60±11.05		
		(1-30)	(1-13)	(5-30)		
		0.003	0.029	0.072		

Therapy	Niv	6(8.1)	6	2	0.000	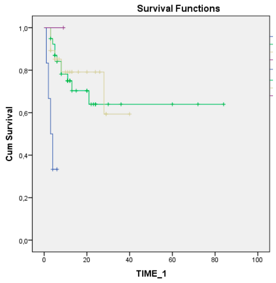
	+Ip	3.50(1.75-4.50)	2.50(1.25-3.75)	5(4-5)		
		3.33±1.75	2.50±1.29	5.00±1.41		
		(1-6)	(1-4)	(4-6)		
	Sun	39(52.7)	11	28		
		12(6-22)	6(4-11)	12.50(11-24)		
		17.41±18.05	7.91±5.39	21.14±19.91		
		(3-84)	(3-21)	(3-84)		
	Paz	28(37.8)	6	22		
		7.50(5-19)	4(3-13)	10.50(5-21)		
		12.18±10.34	8.33±9.83	13.23±10.44		
		(3-40)	(3-28)	(3-40)		
	Sor	1(1.4)	-	1(1.4)		
		9	-	9		
		0.136	0.335	0.243		
MPUL	0	17(23.0)	8(47.1)	9(52.9)	0.571	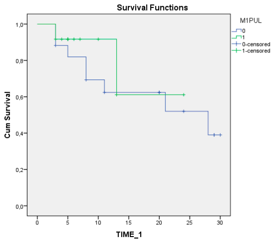
NA		11(6.50-26)	8(3.50-18.50)	20(9.50-29)		
45(60.8)		15.35±10.19	10.88±9.03	19.33±9.92		
		(3-30)	(3-28)	(3-30)		
	1	12(16.2)	2(16.7)	10(83.3)		
		5.50(4.25-12.25)	8	5.50(4.75-10.75)		
		8.17±6.08	8.00±7.07	8.20±6.30		
		(3-24)	(3-13)	(3-24)		
		0.025	0.691	0.009		
MLYM	0	10(13.5)	2(20.0)	8(80.0)	0.197	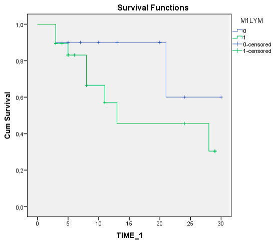
NA		16.50(6.50-21.75)	12	16.50(7.75-23)		
45(60.8)		15.30±8.99	12.00±12.72	16.13±8.77		
		(3-30)	(3-21)	(5-30)		
	1	19(25.7)	8(42.1)	11(57.9)		
		8(4-13)	8(3.50-12.50)	6(4-24)		
		10.84±9.35	9.88±8.14	11.55±10.47		
		(3-29)	(3-28)	(3-29)		
		0.227	0.769	0.316		
MHEP	0	29(39.2)	10	19		
NA		8(5-20.50)	8(3-15)	10(5-24)		
45(60.8)		12.38±9.32	10.30±8.39	13.47±9.81		
		(3-30)	(3-28)	(3-30)		
MOSS	0	21(28.4)	6(28.6)	15	0.335	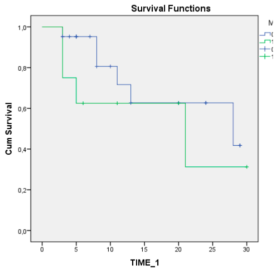
NA		8(5-22)	9.50(6.75-16.75)	8(5-24)		
45(60.8)		12.38±9.26	11.83±8.61	12.60±9.79		
		(3-29)	(3-28)	(3-29)		
	1	8(10.8)	4	4		
		8.50(3.50-20.75)	4(3-17)	15.50(7.25-27.50)		
		12.38±10.11	8.00±8.71	16.75±10.56		
		(3-30)	(3-21)	(6-30)		
		0.999	0.512	0.514		
MOTH		0 28(37.8)				

1	1(1.4)
NA	45(60.8)
